# Combining faecal haemoglobin, iron deficiency anaemia status and age can improve colorectal cancer risk prediction in patients attending primary care with bowel symptoms: a retrospective observational study

**DOI:** 10.1136/gutjnl-2024-334248

**Published:** 2025-03-26

**Authors:** Jayne Digby, Jennifer Nobes, Judith A Strachan, Rebecca McCann, Christopher Hall, Callum G Fraser, Craig Mowat

**Affiliations:** 1 Centre for Research into Cancer Prevention and Screening, University of Dundee, Dundee, Scotland, UK; 2 Division of Respiratory Medicine and Gastroenterology, University of Dundee, Dundee, UK; 3 Department of Blood Sciences, NHS Tayside, Dundee, UK; 4 Health Informatics Centre, University of Dundee, Dundee, Scotland, UK

**Keywords:** COLORECTAL CANCER, PRIMARY CARE, AGEING

## Abstract

**Background:**

In primary care, National Institute for Health and Care Excellence suspected cancer guidelines recommend measuring faecal haemoglobin (f-Hb) if colorectal cancer (CRC) is suspected, with a referral threshold of ≥10 µg Hb/g faeces defining a 3% risk, but most have a normal colonoscopy.

**Objective:**

Examine whether combining f-Hb, patient age and iron-deficient anaemia (IDA) status improves risk prediction.

**Design:**

Retrospective single-centre observational study of symptomatic patients who submitted contemporaneous f-Hb and full blood count (FBC) samples between December 2015 and December 2019. f-Hb was estimated using HM-JACKarc (Hitachi Chemical Diagnostics Systems). Patients were categorised by presence/absence of IDA. Incident CRC was identified via record linkage to the Scottish Cancer Registry. Kaplan-Meier estimates determined cumulative 1-year CRC risk by patient age, f-Hb result and presence of IDA.

**Results:**

Of 34 647 valid f-Hb results retrieved; 7889 (22.8%) had f-Hb≥10 µg Hb/g. Of these, 33 285 samples (96.1%) had associated FBC results of which 3000 (9.0%) had IDA. Overall, 571 incident CRC were recorded. The risk of CRC breached 3% in patients with f-Hb>99 µg Hb/g aged >40 years and reached 30% (19.4–41.0) with f-Hb>99 µg Hb/g in age >55 years plus IDA. 2029 f-Hb results (25.7%) were in the 10–19 µg Hb/g range of which 27 (1.3%) had CRC. In this subgroup, CRC risk did not exceed 3% in patients <85 years and no IDA.

**Conclusion:**

Combining f-Hb, patient age and IDA status improves CRC risk prediction, identifies a low-risk group with f-Hb<20 µg Hb/g and no IDA and could inform revised referral guidance.

**WHAT IS ALREADY KNOWN ON THIS TOPIC**

- In the UK, suspected cancer guidelines recommend referral of symptomatic patients with faecal haemoglobin (f-Hb)≥10 µg Hb/g faeces, but most have a normal colonoscopy.

**WHAT THIS STUDY ADDS**

- Combining patient age, f-Hb and iron-deficient anaemia (IDA) status can stratify colorectal cancer (CRC) risk in patients and guide patient referral and investigation.
- Cumulative 1-year risk of CRC with f-Hb>99 µg Hb/g faeces breached 3% in patients aged >40 years and reached 30% with age >55 years plus IDA.
- Cumulative 1-year CRC risk with f-Hb 10–19 µg Hb/g faeces did not exceed 3% in patients without IDA below 85 years.

**HOW THIS STUDY MIGHT AFFECT RESEARCH, PRACTICE OR POLICY**

- Suspected cancer guidelines could raise the referral threshold to ≥20 µg Hb/g faeces in patients without IDA, and colonoscopy resource could be targeted more effectively.

## Introduction

In the years since the original study,^1^ it has become widely accepted that measurement of faecal haemoglobin concentration (f-Hb) with a faecal immunochemical test (FIT) is a valuable adjunct in the assessment of patients presenting with new onset bowel symptoms in primary care. In the UK, the British Society of Gastroenterology/Association of Coloproctology have produced detailed guidelines which summarise published data^2^ informed by a large meta-analysis.^3^ Diagnostic accuracy studies in primary care using a referral threshold of at least 10 µg Hb/g faeces note remarkably consistent test performance across symptomatic populations with a sensitivity for colorectal cancer (CRC) of~90% and positivity rate of~25%0.^3^ The National Institute for Health and Care Excellence (NICE) has recently updated its guidance for referral for suspected CRC^4 5^ and selected a risk threshold of 3% positive predictive value (PPV) for all cancers to underpin this; in real-world practice, it is more important to be able to predict the proportion of patients with a positive test who have CRC. Both guidelines recommend that adults presenting to primary care with new bowel symptoms or iron-deficient anaemia (IDA) should have an FIT test; patients with a result of at least 10 µg Hb/g faeces should be referred via suspected cancer referral pathways. In the absence of IDA or palpable mass patients with f-Hb<10 µg Hb/g faeces have a very low risk of CRC, and those with f-Hb>400 µg Hb/g faeces had a 54% risk of significant bowel disease.^6^

However, most positive FIT results return intermediate values, and it could be argued that using a single common threshold to determine risk of CRC is a blunt tool. Although increasing f-Hb demonstrates higher risk of CRC,^7^ it is also associated with advancing age and male sex.^8 9^ Therefore, a common dilemma arises when symptomatic patients return an intermediate result: what is their risk (PPV) of underlying CRC and how urgently should they be investigated? However, there is limited published evidence. The potential impact on CRC detection at various f-Hb cut-offs was examined using combined data from three Scottish Health Boards where 1.85% patients with f-Hb in the range 10–19 µg Hb/g faeces who underwent colonoscopy had CRC.^10^ We have also studied the risk of CRC in groups with ‘intermediate’ f-Hb, defined as the range of f-Hb between various chosen reassurance thresholds and a threshold to trigger urgent investigation where PPV for CRC was ≥3%.^11^Although those patients with f-Hb below a threshold of 20 µg Hb/g faeces had a risk of CRC<3%, 9 of 18 patients below this threshold with CRC had IDA and it was concluded that patients with f-Hb<20 µg Hb/g faeces and IDA should still undergo further investigation. Combining FIT with other routinely collected data to improve diagnostic accuracy has previously been attempted. The FAST (Faecal haemoglobin, Age and Sex Test) score combined f-Hb with age and gender,^12^ but a subsequent study failed to replicate the findings and suggested further work was required.^13^ A systematic review of risk prediction models for colorectal neoplasia in symptomatic patients reported that those using a combination of FIT with other variables were generally good but concluded that further studies were required.^14^ Our group attempted to construct a risk prediction model combining f-Hb, age, gender, IDA and systemic immune inflammation index,^15^ but this did not result in convincing improvement in test performance at the 10 µg Hb/g faeces threshold. As with the FAST score, sensitivity for CRC could be improved but at the expense of high positivity rate and increased colonoscopy demand. We concluded that other approaches should be explored.

More recently, published results from a large cohort of symptomatic patients completing FIT and a full blood count (FBC) in primary care settings in Nottingham, England found that patients aged <70 years old with f-Hb<100 µg Hb/g and no IDA had a CRC risk of <3% in the following year.^16^

In this retrospective study, we aimed to explore the f-Hb distribution of those with test results >10 µg Hb/g faeces in our population of symptomatic patients. We then aimed to examine the prevalence of CRC across a spectrum of f-Hb results and determine whether the prevalence of CRC could be further stratified by patient age, and the presence or absence of IDA.

## Methods

NHS Tayside serves a population of 414 000 and is the fourth largest healthcare provider in Scotland. From December 2015, quantitative FIT analysis has been available to primary care (general practitioner (GP)) surgeries across the region to assist in the clinical assessment of patients presenting with new onset bowel symptoms. Surgeries were provided with written information detailing the rationale for measuring f-Hb, and FIT kits comprising one specimen collection device (Hitachi Chemical Diagnostics Systems, Tokyo, Japan) and a pictorial patient instruction sheet. The full methodology is already described.^6^ In brief, f-Hb was measured using a single HM-JACKarc analyser (Hitachi Chemical Diagnostics Systems, Tokyo, Japan) with an analytical measurement range of 7–400 µg Hb/g faeces. Samples with results above the upper measurement limit were not diluted and re-assayed but reported as ≥400 µg Hb/g faeces. GPs were advised to request a simultaneous FBC; electronic laboratory order communication software promotes this by providing a ‘colorectal bundle’ of tests which can be requested with one click—comprising FIT, FBC and renal function (to ensure safe prescription of oral bowel preparation if colonoscopy subsequently booked). Patients with f-Hb≥10 µg Hb/g faeces were recommended to be referred for further investigation, exactly as subsequently recommended in NICE DG56.^4^

Patients referred to endoscopy were investigated within 6 weeks of referral. All findings were recorded electronically on the endoscopy reporting system by the endoscopists. The diagnoses of CRC were confirmed by a gastrointestinal pathologist.

All f-Hb results generated since the inception of the service in December 2015 until December 2019 were included to eliminate any bias and were retrospectively retrieved from the laboratory information management system (LIMS). Numerical f-Hb were grouped into 10 µg Hb/g faeces ranges from 10 to 399 µg Hb/g faeces, and thereafter ≥400 µg Hb/g faeces. Anonymised record-linkage was performed between all study patients and the Scottish Cancer Registry, which was complete for cancer diagnoses registered up to June 2022. If there were more than one f-Hb result from a single patient, the FIT result most closely chronologically associated with a CRC diagnosis was included in the analysis and consequently (for those patients who had submitted more than one sample) there would be no violations of the assumption of independence. Follow-up was standardised to 2 years from the date of FIT for all patients. This enabled analysis of CRC incidence and calculation of PPV by f-Hb threshold as a measure of CRC risk. Further linkage was performed with electronic patient records to identify the FBC associated with the FIT request. Patients were classified according to whether IDA was present, defined pragmatically as Hb<130 g/L in men and <120 g/L in women alongside mean corpuscular volume (MCV)<85 fL (as per the local population-based reference interval).^17^ R within R Studio statistical software (V.4.1.3, RStudio Team (2022). RStudio: Integrated Development for R. RStudio, PBC, Boston, Massachusetts, USA) was used for all calculations and generation of heat maps. We used the survival package to calculate the 1-year Kaplan-Meier survival by each subgroup defined by f-Hb, age and IDA categories. 1-year cumulative CRC risk with 95% CIs was calculated as one minus the 1-year Kaplan-Meier survival. Using the *ggplot2* package, this data was presented as heat maps, replicating the work of Crooks *et al*^16^ in a separate population.

### Patient and public involvement

There was no patient or public involvement in this study.

## Results

### Faecal haemoglobin concentration distributions

From December 2015 to December 2019, a total of 35 587 samples for FIT analysis were received from 29 605 individual patients assessed in primary care; median age of 65 years, of whom 57.9% were women. 4502 patients completed two, 739 completed three, 131 completed four, 25 completed five and four completed six FITs within this time. Overall, 732 (2.1%) were unsuitable for analysis, most commonly due to faecal contamination on the outside of the collection device. A further 208 patients who were aged under 18 years were excluded from the analysis.

**Figure 1:**
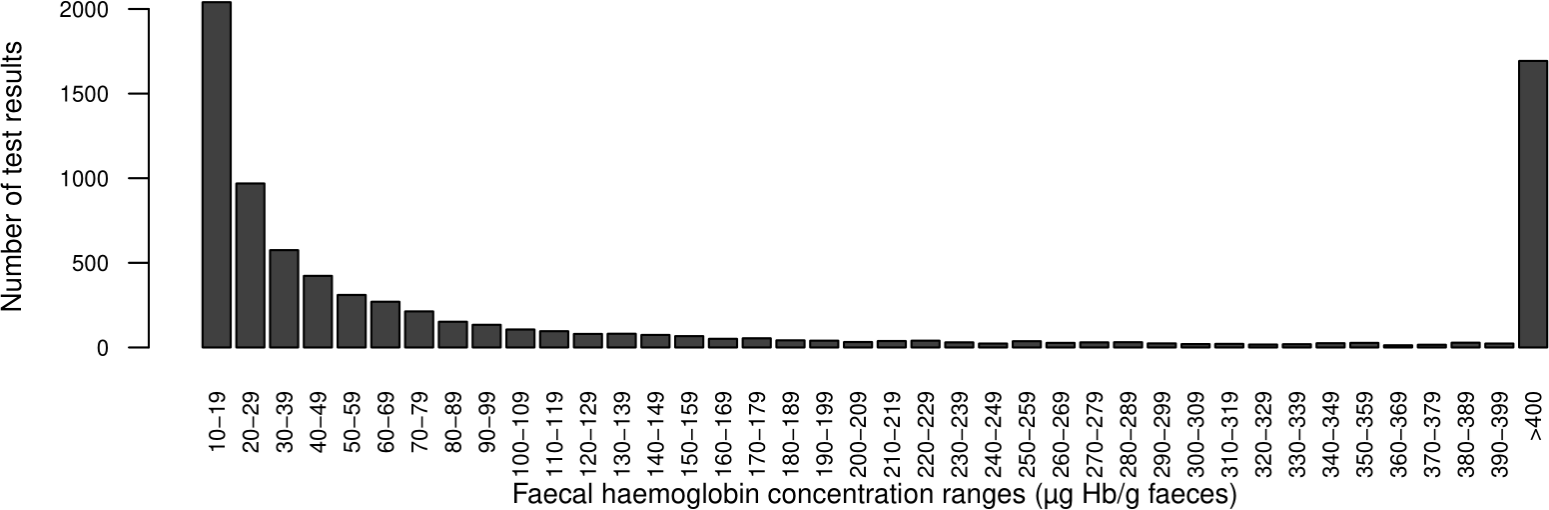
Distribution of faecal haemoglobin concentration results ≥10 µg Hb/g faeces in symptomatic patients assessed in primary care (n=7889).

Of the remaining 34 647 samples which provided a valid f-Hb result (57.9% women), 7889 (22.7%) were ≥10 µg Hb/g faeces. The distribution of the positive f-Hb results is shown in 10 µg Hb/g faeces ranges in figure 1. Two-thirds of these results were below 120 µg Hb/g faeces (10–119 µg Hb/g faeces; 5263/7889: 66.7%). 2029 of results with f-Hb≥10 µg Hb/g faeces were in the 10–19 µg Hb/g faeces range (25.7%). Only 996/7889 (12.6%) were in the 120–399 µg Hb/g faeces range, but 1688 (21.4%) had f-Hb≥400 µg Hb/g faeces.

A total of 571 CRCs were diagnosed within a homogeneous follow-up time of 2 years after the FIT result (1.6%). The PPV for CRC was 0.3% in patients with f-Hb<10 µg Hb/g faeces. A total of 2029 test results were within the 10–19 µg Hb/g faeces range identified of which 27 (1.3%) had CRC. The 3% threshold outlined by the NICE guidance for suspected cancer was not surpassed until the f-Hb range of 30–39 µg Hb/g faeces (22/574: 3.8%). Overall, the PPV for CRC was 6.2% in patients with f-Hb≥10 µg Hb/g faeces, 7.9% (462/5860) in patients with f-Hb≥20 µg Hb/g faeces, rising to 12.9% in patients with f-Hb>99 µg Hb/g faeces. The highest proportion of CRC, 16.1%, was identified in patients with f-Hb≥400 µg Hb/g faeces (272/1688) (data on file).

### CRC risk by faecal haemoglobin threshold and patient age

As expected, factoring in patient age modified the PPV for CRC. At each f-Hb threshold, PPV for CRC increased with increasing patient age. The 3% risk was breached for the first time in the 20–39.9 µg Hb/g faeces category, but only in patients older than 85 years. In patients older than 40 years, it was breached in the >99 µg Hb/g faeces category. The highest risk (18.4%) was seen in patients with f-Hb>99 in the age category 71–85 years. Online supplemental table S1

A total of 33 285/34 647 (96.1%) had a valid simultaneous FBC, with 3000 (9.0%) having IDA. The number of patients with FBC available and prevalence of IDA by age and sex is shown in table 1. In both sexes, the prevalence of IDA increased with age and was greater in women within all age brackets. The highest prevalence of IDA (14.1%) was within women aged 41–55 years, which may relate to peri-menopausal menorrhagia.

**Table 1:** Number of patient samples with full blood count (FBC) available and the percentage with iron deficiency anaemia (IDA) by age and sex

	Females		Males		Total	
	**with FBC**	**with IDA (%**)	**with FBC**	**with IDA (%**)	**with FBC**	**with IDA (%**)
Age (years)						
18–40	2013	151 (7.5)	1490	30 (2.0)	3503	181 (5.2)
41–55	3814	537 (14.1)	2290	82 (3.6)	6104	619 (10.1)
56–70	5613	495 (8.8)	4183	285 (6.8)	9796	780 (8.0)
71–85	6468	720 (11.1)	4757	403 (8.5)	11 225	1123 (10.0)
85+	1499	206 (13.7)	1158	91 (7.9)	2657	297 (11.2)
Total	19 407	2109 (10.9)	13 878	891 (6.4)	33 285	3000 (9.0)

Of those patients subsequently diagnosed with CRC, 567/571 had an FBC checked at the time of referral. Online supplemental table S2 shows the number of CRC diagnosed according to age, f-Hb category and the presence or absence of IDA. Overall, 138/3000 (4.6%) patients with IDA had CRC compared with 429/30 285 (1.4%) without IDA. As expected, factoring in the presence of IDA in addition to patient age and f-Hb threshold modified the PPV for CRC. In patients with f-Hb<10 µg Hb/g faeces and IDA, the PPV for CRC increased from 0.3% to 0.7% overall but clearly did not meet the 3% threshold. The 3% risk was breached for the first time in the 10–19.9 µg Hb/g faeces category, but only in patients older than 70 years. In patients with f-Hb>99 µg Hb/g faeces, the overall risk of CRC increased from 10% to 26.9% in the presence of IDA. In the absence of IDA, overall, the 3% threshold was not surpassed until the f-Hb was >99 µg Hb/g faeces. In patients aged 18–40 years in this cohort, the risk of CRC was highest (2.3%) in patients with f-Hb>99 and no IDA, but numbers are small.

### 1-year cumulative CRC risks by age, f-Hb and iron deficiency anaemia status

**Figure 2:**
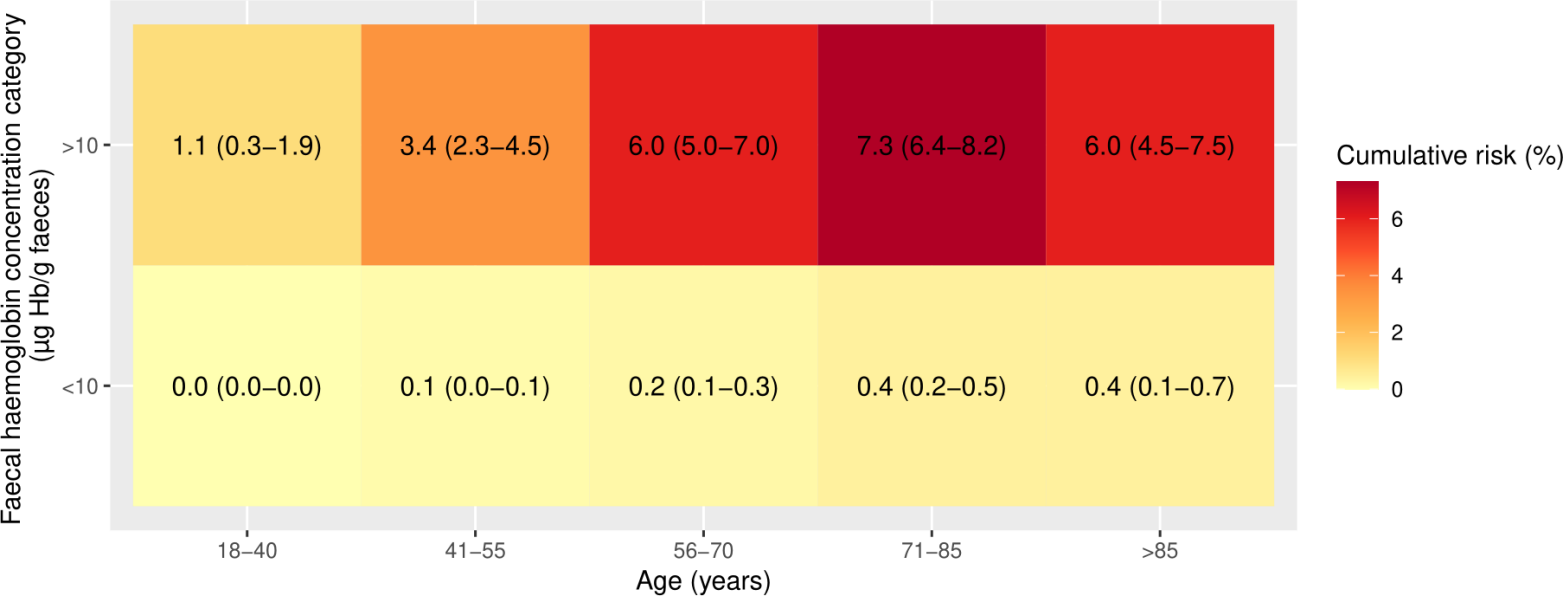
Heat map of cumulative 1-year colorectal cancer risk (95% CI) by categories of age and faecal haemoglobin concentration dichotomised at 10 µg Hb/g faeces.

**Figure 3:**
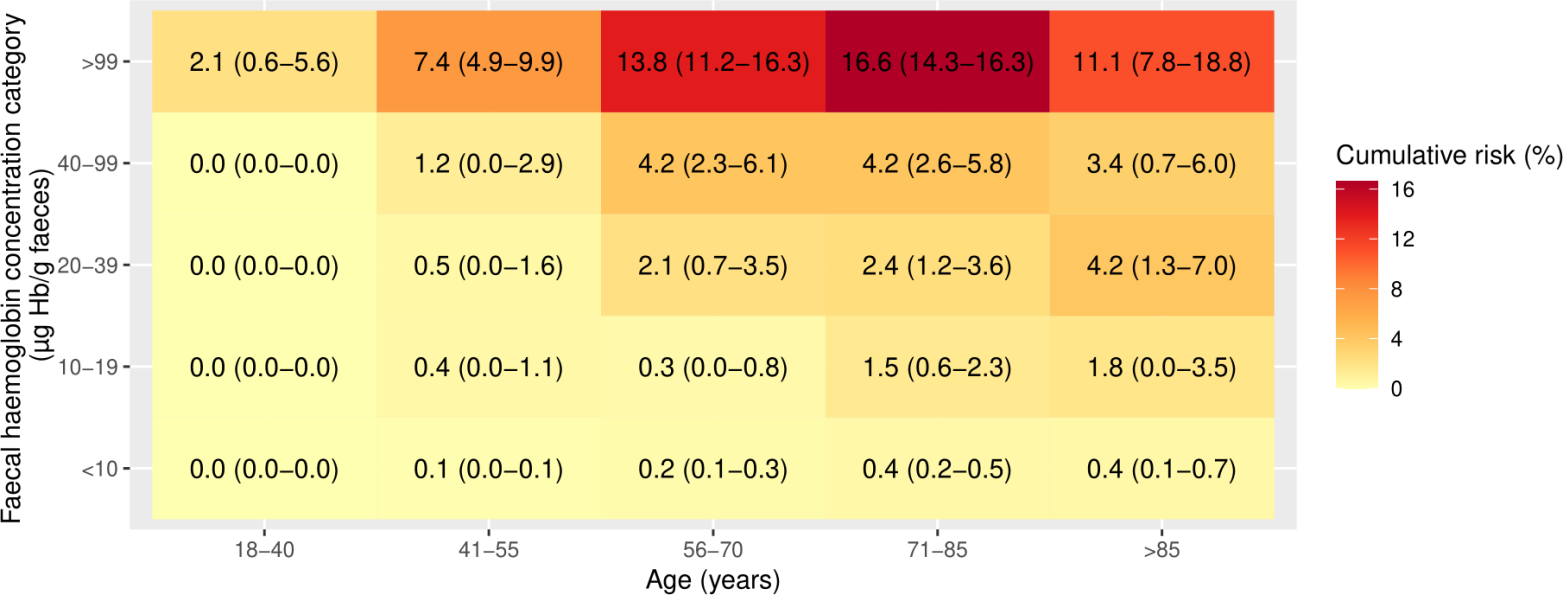
Heat map of cumulative 1-year colorectal cancer risk (95% CI) by categories of age and faecal haemoglobin concentration.

**Figure 4:**
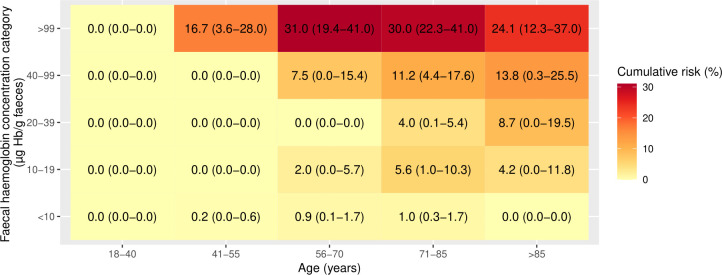
Heat map of cumulative 1-year colorectal cancer risk (95% CI) in those with iron-deficiency anaemia, by categories of age and faecal haemoglobin concentration.

**Figure 5:**
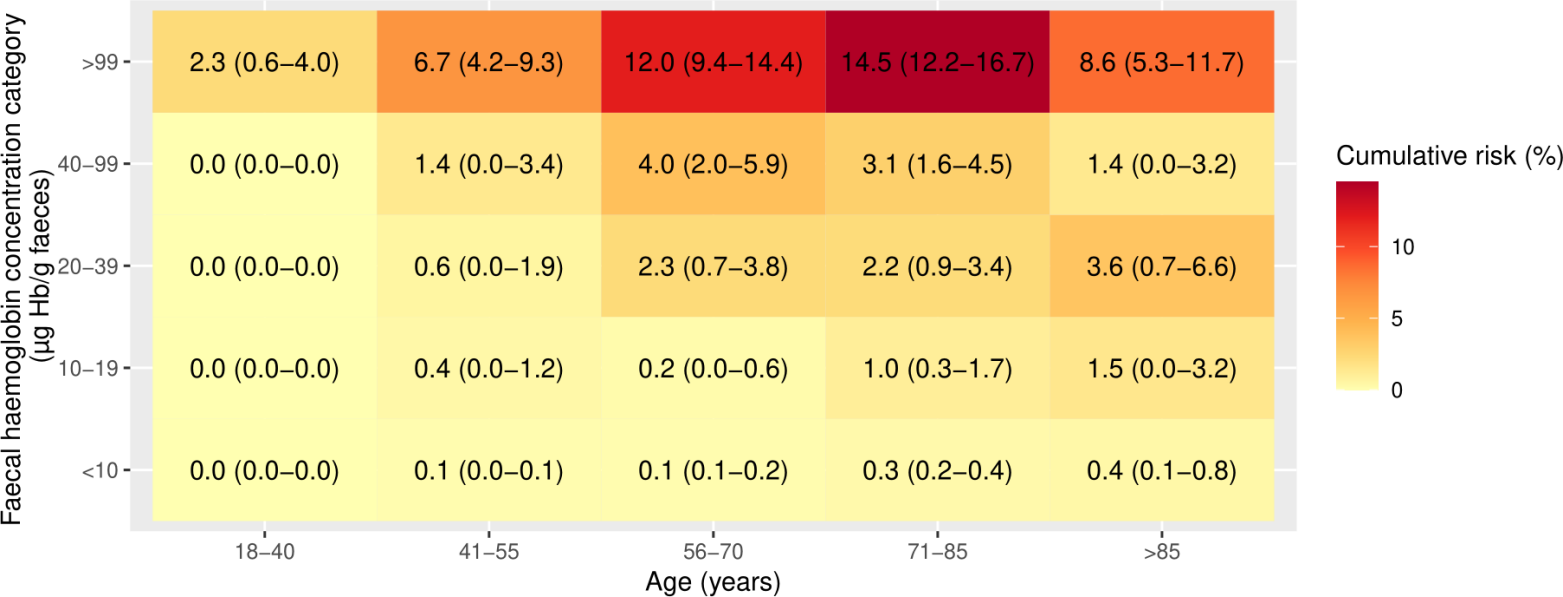
Heat map of cumulative 1-year colorectal cancer risk (95% CI) in those without iron-deficiency anaemia, by categories of age and faecal haemoglobin concentration.

Using the NICE recommended threshold of f-Hb≥10 µg Hb/g faeces, all patients except those aged 18–40 years had a 1-year cumulative CRC risk >3% (figure 2) and the risk of CRC increased with increasing age up to age 85 years. When 1-year cumulative CRC risk was assessed by further refined categories of f-Hb, the risk of CRC was highest in those with f-Hb≥100 µg Hb/g faeces and generally increased with increasing age (figure 3). In those aged 55 years or younger, the 3% threshold was not reached in any f-Hb category <100 µg Hb/g faeces. Furthermore, the risk threshold of 3% was not surpassed for those with f-Hb 10–19 µg Hb/g faeces within any age group. The 1-year cumulative CRC risk in patients with f-Hb 10–19 µg Hb/g faeces and IDA may exceed 3% (based on the upper 95% CI bound of 5.7%) in patients aged >55 years (figure 4). In patients without IDA, the highest 1-year cumulative CRC risk in those with f-Hb 10–19 µg Hb/g faeces was in those aged over 85 years where the risk was 1.5 (95% CI 0.0 to 3.2), (figure 5). CRC risk in patients with IDA was generally double that observed in those without IDA at the equivalent age and f-Hb range.

## Discussion

In this large retrospective single-centre study we have demonstrated that the CRC risk in symptomatic patients attending primary care can be refined by combining f-Hb result with patient age and IDA status. In patients with f-Hb<10 µg Hb/g faeces the overall cumulative 1-year risk of CRC was less than 1% irrespective of age or IDA status. In the absence of IDA, patients with f-Hb in the 10–19 µg Hb/g faeces range (which accounts for one quarter of all positive test results using the threshold of f-Hb≥10 µg) had a CRC risk that peaked at 1.5% (95% CI 0.0% to 3.2%) in patients over 85 years. Thereafter, the risk of CRC increased with age and f-Hb to a maximum of 14.5% (95% CI 12.2% to 14.5%) in patients aged 71–85. We have confirmed that the presence of IDA is associated with an increased risk of CRC, and that this increased with patient age and f-Hb concentrations.

Importantly, our results validate the findings from Crooks *et al* that patients with f-Hb in the 10–19 µg Hb/g faeces range and no IDA have a 1-year cumulative CRC risk which is below 3%. This compares very favourably with the 3-year unadjusted post-colonoscopy CRC rate of 7.4% in England.^18^ These findings have important ramifications for stakeholders involved in the recommended use of FIT in patients with new onset colorectal symptoms and could impact on clinical decision-making for many thousands of patients. In Scotland alone, more than 200 000 symptomatic FIT tests were submitted in 2023 (data supplied by NHS Scotland, Centre for Sustainable Delivery). In healthcare systems where colonoscopy resource is finite, directing patients to investigation efficiently is a priority, and this accumulating evidence suggests it would be appropriate to raise the recommended f-Hb threshold for referral from 10 to 20 µg Hb/g faeces in those without IDA and enable colonoscopy resource to be re-allocated, perhaps to the bowel screening programme.

The threshold of ≥10 µg Hb/g faeces, as advocated in the NICE DG56 guideline,^4^ was based on the evidence collated to underpin the guideline.^19^ This evidence was rather sparse and relied mainly on two small studies performed in Scotland, using different FIT analytical systems, and receiver operating characteristic analysis which gave the optimum balance between sensitivity and specificity for CRC, high-risk adenoma and inflammatory bowel disease as 10 µg Hb/g faeces.^10 11^ This was supported by a larger study in Spain.^12^ Since the wider adoption of FIT in the assessment of patients presenting with symptoms, interest had been focused on the use of low f-Hb thresholds, approaching the limit of detection and limit of quantitation of the FIT analytical system applied.^20^ These include 2 µg Hb/g faeces,^21^ 4 µg Hb/g faeces^22 23^ and 7 µg Hb/g faeces.^24^ These f-Hb thresholds were studied because of the concern that CRC would be missed using higher thresholds, even such as 10 µg Hb/g faeces. Unsurprisingly, CRC detection did improve at lower f-Hb thresholds with higher clinical sensitivity, but at the expense of higher positivity, lower positive predictive value (due to the higher number of false positive results) and greater endoscopy demand. Although an early study in Spain used a threshold of 20 µg Hb/g faeces,^25^ there have been few studies on higher f-Hb thresholds, particularly those just above 10 µg Hb/g faeces.

The strengths of this study are the large patient population, the robust CRC diagnosis using record-linkage with the Scottish Cancer Registry and the homogenous follow-up time of 2 years. An additional strength is the fact that we have replicated the findings of Crooks *et al*; furthermore, their f-Hb data was generated using a different analyser (OC-Sensor platform (Eiken Chemical)) which until now has always been perceived as a barrier when comparing patient outcomes across different cohorts. Also, we have focused our summary of risk prediction of CRC using PPV, because it is used by NICE and readily understood by clinicians, considers baseline risk factors and provides a measure of the reliability of a positive test which is of greater clinical value and could be applied across differing populations with similar baseline risk.^26^ A key limitation of this study is that the numbers of patients with CRC and IDA are small in some categories of f-Hb and age, meaning that our analysis of CRC risk in these groups generated wide CIs and should be interpreted with caution. This is also apparent in the Crooks *et al* paper, and it is not clear how many patients had CRC and IDA in their cohort. It is noteworthy that we used the presence of microcytic anaemia as a proxy for IDA as this data was readily available, and we did not use other biochemical indices. However, the catchment population in this part of Scotland is predominantly Caucasian, and thalassaemia traits are rare. We were not able to exclude other causes of IDA, such as menorrhagia in pre-menopausal women and would recommend that GPs continue to interpret such results in the context of the individual patient in front of them. Another important limitation is that data on other significant bowel disease including high-risk adenoma as a potential precursor to CRC, and inflammatory bowel disease, were not available. However, our findings do allow assessment of the positive predictive value and 1-year cumulative risk of CRC in increasing ranges of f-Hb for comparison with the 3% threshold outlined by the NICE guidelines for suspected cancer.^5^ Finally, an elevated f-Hb is associated with increased all-cause mortality,^27^ but we did not record-link our cohort to the deaths register. We have previously reported that the median time from symptomatic FIT test to CRC diagnosis in this study cohort was only 23 days,^28^ and while we are not aware of any patient deaths from non-CRC causes within the 2-year follow-up period this is a limitation. Theoretically, this could result in an overestimated risk of CRC in elderly subjects, particularly those over 85 years. However, we do not believe this would change the conclusions of this study.

As we have demonstrated, a ‘positive’ f-Hb result produced by the laboratory in isolation does not convey a precise risk of CRC. Modern diagnostic laboratory services have the capability of adding clinical value^29^ beyond that of generating an accurate estimation of f-Hb concentration and could provide a solution to this problem. Increasingly, artificial intelligence (AI) approaches have been described, varying in complexity from automated ‘decision-tree’ systems—sometimes called ‘expert systems’—which replicate human interpretation, to machine learning (ML) techniques.^30^ There are multiple limitations and barriers to the use of ML in healthcare^31^ and, as discussed by Mülder *et al*, ML approaches may not outperform less complex logistic regression models, especially where there are few included variables.^32^ However, simpler algorithm-led AI approaches are already part of routine practice and may be well-suited to this scenario in which limited clinical information (f-Hb, IDA and age) could be combined to provide more information than just reporting the f-Hb alone. One example of a similar system is the intelligent liver function testing pathway in NHS Tayside in which liver blood test results are combined with additional clinical information, allowing automated generation of a predicted diagnosis and associated management plan.^33^ This approach could be used to improve the clinical utility of FIT in the context of suspected CRC; demographic information (patient age and sex), f-Hb concentration, haemoglobin and MCV could be combined using algorithms within the LIMS to provide a more precise estimate of CRC risk, based on the heat maps developed above and the prior work from Crooks *et al*. This would allow a semi-personalised risk of CRC to be reported back to the requesting clinician—with further information and safety-netting advice available electronically—potentially aiding discussion with the patient and helping to guide the need for onward investigation (with reference to the 3% NICE threshold).

In summary, for endoscopy services faced with a demand which outstrips capacity, we have shown that CRC risk can be stratified by f-Hb, age and IDA status which can inform prioritisation for rapid investigation and target colonoscopy more efficiently to those who would benefit most.^34^ Furthermore, we believe that the threshold for f-Hb used in practice to trigger referral for colonoscopy in patients presenting with symptoms could be safely raised to ≥20 µg Hb/g faeces provided safety-netting with FBC analysis (to identify IDA) and repeat FIT for persisting bowel symptoms is in place. This strategy would reduce the proportion of patients referred for investigation while still identifying those patients aged >55 years with IDA that should be investigated. Future studies could focus on laboratory software implementation of intelligent f-Hb thresholds tailored to maintain a 3% CRC risk according to age and IDA status using much larger cohorts.

## Supplementary material

10.1136/gutjnl-2024-334248 online supplemental file 1

10.1136/gutjnl-2024-334248 online supplemental file 2

## Data Availability

Data are available upon reasonable request.
