# Supplementary material for: Combining faecal haemoglobin, iron deficiency anaemia status and age can improve colorectal cancer risk prediction in patients attending primary care with bowel symptoms: a retrospective observational study
Source: Gut. 2025 Mar 26;74(9):e334248. doi: 10.1136/gutjnl-2024-334248 (PMC12418543; doi:10.1136/gutjnl-2024-334248)
Supplement: online supplemental file 1 [file gutjnl-74-9-s001.docx]

**Supplementary Material**

**Supplementary Table s1**: **Number of colorectal cancer (CRC) cases in a two-year follow-up period and the associated percentage of patient samples diagnosed (Positive Predictive Value) according to age and faecal haemoglobin**

|  |  | **Faecal haemoglobin concentration category (µg Hb/g faeces)** | | | | | | | | | | | | | | |
| --- | --- | --- | --- | --- | --- | --- | --- | --- | --- | --- | --- | --- | --- | --- | --- | --- |
|  |  | **<10** | | | **10-19.9** | | | **20-39.9** | | | **40-99** | | | **>99** | | |
|  |  | **n** | **CRC** | **%** | **n** | **CRC** | **%** | **n** | **CRC** | **%** | **n** | **CRC** | **%** | **n** | **CRC** | **%** |
| **All patient samples** | |  |  |  |  |  |  |  |  |  |  |  |  |  |  |  |
| **Age (years)** | |  |  |  |  |  |  |  |  |  |  |  |  |  |  |  |
|  | **18-40** | 3093 | 0 | 0.0 | 124 | 0 | 0.0 | 90 | 0 | 0.0 | 107 | 0 | 0.0 | 332 | 7 | 2.1 |
|  | **41-55** | 5462 | 4 | 0.1 | 270 | 1 | 0.4 | 184 | 2 | 1.1 | 164 | 2 | 1.2 | 420 | 32 | 7.6 |
|  | **56-70** | 8084 | 23 | 0.3 | 582 | 4 | 0.7 | 419 | 10 | 2.4 | 426 | 18 | 4.2 | 719 | 100 | 13.9 |
|  | **71-85** | 8364 | 45 | 0.5 | 827 | 18 | 2.2 | 657 | 19 | 2.9 | 617 | 27 | 4.4 | 1006 | 185 | 18.4 |
|  | **85+** | 1755 | 10 | 0.6 | 226 | 4 | 1.8 | 190 | 11 | 5.8 | 179 | 7 | 3.9 | 350 | 42 | 12.0 |
|  | **Total** | 26758 | 82 | 0.3 | 2029 | 27 | 1.3 | 1540 | 42 | 2.7 | 1493 | 54 | 3.6 | 2827 | 366 | 12.9 |

**Supplementary Table s2. Number of colorectal cancer (CRC) cases in a two-year follow-up period and the associated percentage of patients diagnosed (Positive Predictive Value) according to age, faecal haemoglobin concentration category and iron deficiency anaemia (IDA) status.**

|  |  | **Faecal haemoglobin concentration category (µg Hb/g faeces)** | | | | | | | | | | | | | | |
| --- | --- | --- | --- | --- | --- | --- | --- | --- | --- | --- | --- | --- | --- | --- | --- | --- |
|  |  | **<10** | | | **10-19.9** | | | **20-39.9** | | | **40-99** | | | **>99** | | |
|  |  | **n** | **CRC** | **%** | **n** | **CRC** | **%** | **n** | **CRC** | **%** | **n** | **CRC** | **%** | **n** | **CRC** | **%** |
| **All patients** | |  |  |  |  |  |  |  |  |  |  |  |  |  |  |  |
| **With IDA** | | | | |  | | |  | | |  | | |  | | |
| **Age (years)** | |  |  |  |  |  |  |  |  |  |  |  |  |  |  |  |
|  | **18-40** | 138 | 0 | 0.0 | 12 | 0 | 0.0 | 5 | 0 | 0.0 | 7 | 0 | 0.0 | 19 | 0 | 0.0 |
|  | **41-55** | 522 | 1 | 0.2 | 26 | 0 | 0.0 | 18 | 0 | 0.0 | 17 | 0 | 0.0 | 36 | 6 | 19.2 |
|  | **56-70** | 566 | 5 | 0.9 | 51 | 1 | 2.0 | 52 | 0 | 0.0 | 40 | 3 | 7.5 | 71 | 22 | 31.0 |
|  | **71-85** | 703 | 9 | 1.3 | 89 | 6 | 6.7 | 92 | 5 | 5.4 | 89 | 10 | 11.2 | 150 | 48 | 32.0 |
|  | **85+** | 163 | 1 | 0.6 | 24 | 1 | 4.2 | 23 | 2 | 8.7 | 29 | 4 | 13.8 | 58 | 14 | 24.1 |
|  | **Total** | 2092 | 16 | 0.7 | 202 | 8 | 4.0 | 190 | 7 | 3.7 | 182 | 17 | 9.3 | 334 | 90 | 26.9 |
| **No IDA** | |  | | |  | | |  | | |  | | |  | | |
| **Age (years)** | |  |  |  |  |  |  |  |  |  |  |  |  |  |  |  |
|  | **18-40** | 2736 | 0 | 0.0 | 107 | 0 | 0.0 | 80 | 0 | 0.0 | 97 | 0 | 0.0 | 302 | 7 | 2.3 |
|  | **41-55** | 4575 | 3 | 0.1 | 240 | 1 | 0.4 | 159 | 2 | 1.2 | 140 | 2 | 1.4 | 371 | 26 | 7.0 |
|  | **56-70** | 7132 | 18 | 0.3 | 518 | 3 | 0.6 | 353 | 9 | 2.5 | 379 | 15 | 4.0 | 634 | 77 | 12.1 |
|  | **71-85** | 7396 | 35 | 0.5 | 720 | 12 | 1.6 | 556 | 14 | 2.5 | 525 | 17 | 3.2 | 905 | 136 | 15.0 |
|  | **85+** | 1558 | 9 | 0.6 | 197 | 3 | 1.5 | 166 | 9 | 5.4 | 148 | 3 | 2.0 | 291 | 28 | 9.6 |
|  | **Total** | 23397 | 65 | 0.3 | 1782 | 19 | 1.1 | 1314 | 34 | 2.6 | 1289 | 37 | 2.9 | 2503 | 274 | 10.0 |
