# Supplementary material for: Combining faecal haemoglobin, iron deficiency anaemia status and age can improve colorectal cancer risk prediction in patients attending primary care with bowel symptoms: a retrospective observational study
Source: Gut. 2025 Mar 26;74(9):e334248. doi: 10.1136/gutjnl-2024-334248 (PMC12418543; doi:10.1136/gutjnl-2024-334248)
Supplement: online supplemental file 2 [file gutjnl-74-9-s002.pptx]

## Slide 1
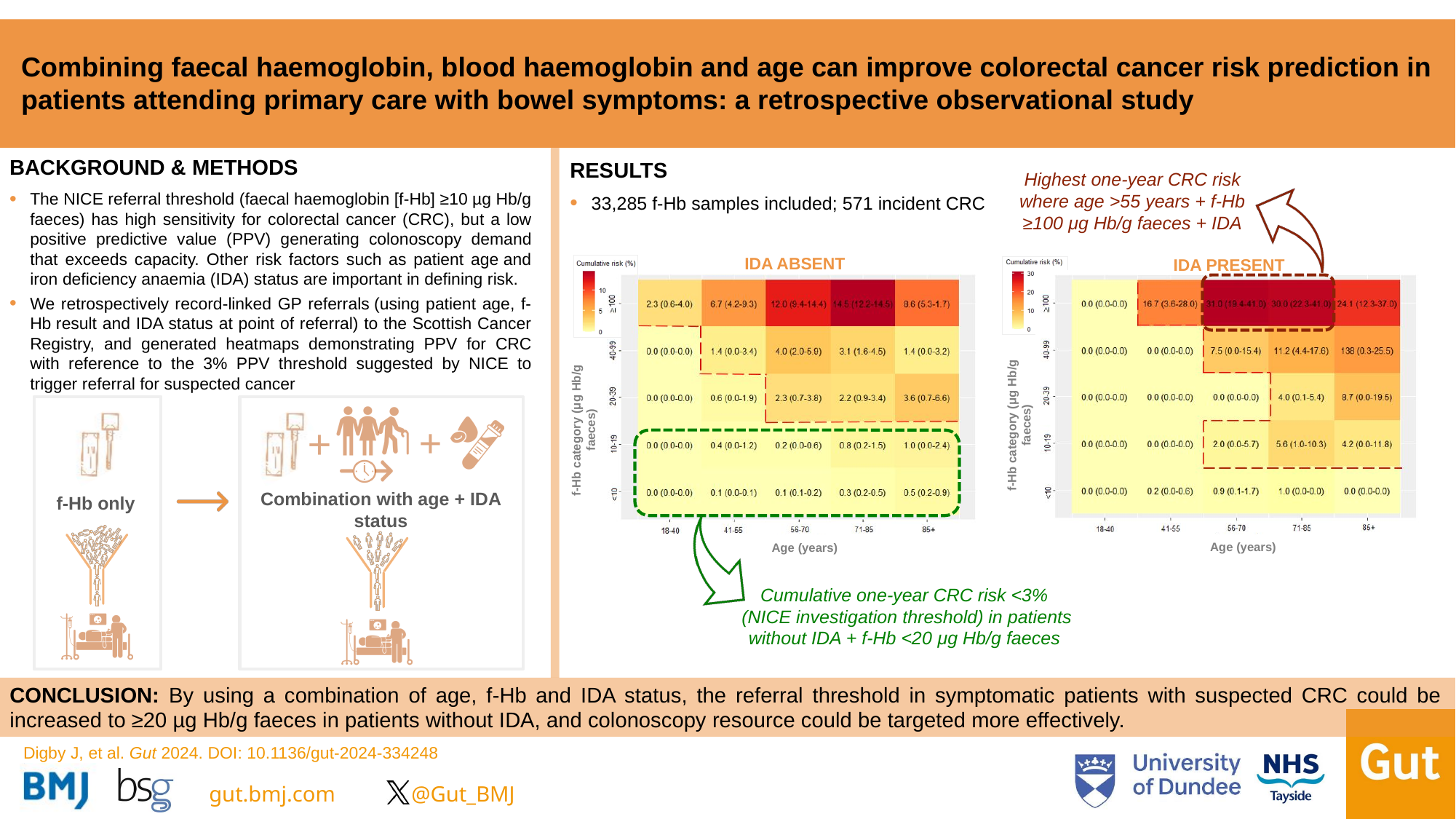

Combining faecal haemoglobin, blood haemoglobin and age can improve colorectal cancer risk prediction in patients attending primary care with bowel symptoms: a retrospective observational study
BACKGROUND & METHODS
The NICE referral threshold (faecal haemoglobin [f-Hb] ≥10 µg Hb/g faeces) has high sensitivity for colorectal cancer (CRC), but a low positive predictive value (PPV) generating colonoscopy demand that exceeds capacity. Other risk factors such as patient age and iron deficiency anaemia (IDA) status are important in defining risk.
We retrospectively record-linked GP referrals (using patient age, f-Hb result and IDA status at point of referral) to the Scottish Cancer Registry, and generated heatmaps demonstrating PPV for CRC with reference to the 3% PPV threshold suggested by NICE to trigger referral for suspected cancer
RESULTS
33,285 f-Hb samples included; 571 incident CRC
Highest one-year CRC risk where age >55 years + f-Hb ≥100 μg Hb/g faeces + IDA
IDA ABSENT
IDA PRESENT
Combination with age + IDA status
f-Hb only
f-Hb category (μg Hb/g faeces)
f-Hb category (μg Hb/g faeces)
Age (years)
Age (years)
Cumulative one-year CRC risk <3%
(NICE investigation threshold) in patients without IDA + f-Hb <20 μg Hb/g faeces
CONCLUSION: By using a combination of age, f-Hb and IDA status, the referral threshold in symptomatic patients with suspected CRC could be increased to ≥20 µg Hb/g faeces in patients without IDA, and colonoscopy resource could be targeted more effectively.
Digby J, et al. Gut 2024. DOI: 10.1136/gut-2024-334248
gut.bmj.com
@Gut_BMJ
